# Remodeling of Cell Wall Components in Root Nodules and Flower Abscission Zone under Drought in Yellow Lupine

**DOI:** 10.3390/ijms23031680

**Published:** 2022-01-31

**Authors:** Emilia Wilmowicz, Agata Kućko, Juan De Dios Alché, Grażyna Czeszewska-Rosiak, Aleksandra Bogumiła Florkiewicz, Małgorzata Kapusta, Jacek Karwaszewski

**Affiliations:** 1 Chair of Plant Physiology and Biotechnology, Nicolaus Copernicus University, Lwowska 1 Street, 87-100 Toruń, Poland; wiktoria@umk.pl (G.C.-R.); a.florkiewicz21@gmail.com (A.B.F.); 293686@stud.umk.pl (J.K.); 2 Department of Plant Physiology, Institute of Biology, Warsaw University of Life Sciences-SGGW (WULS-SGGW), Nowoursynowska 159 Street, 02-776 Warsaw, Poland; agata_kucko@sggw.edu.pl; 3 Plant Reproductive Biology and Advanced Microscopy Laboratory, Department of Biochemistry, Cell and Molecular Biology of Plants, Estación Experimental del Zaidín, Spanish National Research Council (CSIC), Profesor Albareda 1, E-18008 Granada, Spain; juandedios.alche@eez.csic.es; 4 Department of Plant Cytology and Embryology, University of Gdańsk, Wita Stwosza 59 Street, 80-308 Gdańsk, Poland; malgorzata.kapusta@biol.ug.edu.pl

**Keywords:** abscission zone, arabinan, cell wall, drought, extensins, galactans, root nodules, xyloglucans, yellow lupine, yielding

## Abstract

We recently showed that yellow lupine is highly sensitive to soil water deficits since this stressor disrupts nodule structure and functioning, and at the same time triggers flower separation through abscission zone (AZ) activation in the upper part of the plant. Both processes require specific transformations including cell wall remodeling. However, knowledge about the involvement of particular cell wall elements in nodulation and abscission in agronomically important, nitrogen-fixing crops, especially under stressful conditions, is still scarce. Here, we used immuno-fluorescence techniques to visualize dynamic changes in cell wall compounds taking place in the root nodules and flower AZ of *Lupinus luteus* following drought. The reaction of nodules and the flower AZ to drought includes the upregulation of extensins, galactans, arabinans, xylogalacturonan, and xyloglucans. Additionally, modifications in the localization of high- and low-methylated homogalacturonans and arabinogalactan proteins were detected in nodules. Collectively, we determined for the first time the drought-associated modification of cell wall components responsible for their remodeling in root nodules and the flower AZ of *L. luteus*. The involvement of these particular molecules and their possible interaction in response to stress is also deeply discussed herein.

## 1. Introduction

Two particular processes are of paramount importance for the yielding of leguminous plants, including yellow lupine (*Lupinus luteus* L.). On the one hand, proper functioning of root nodules, which is guaranteed by the occurrence of a symbiotic plant–bacteria relationship and enables atmospheric nitrogen (N_2_) fixation. On the other hand, the development and maintenance of flowers in the maternal plant and as a consequence the formation of protein-enriched pods. Flavonoids, synthesized in the roots of lupine and excreted to the rhizosphere, induce bacteria to biosynthesize Nod factors of the lipo-chitooligosaccharide (LCO) type which trigger nodulation [1]. In developed root nodules, N_2_ fixation is catalyzed by nitrogenase, the activity of which strongly depends on the action of leghemoglobin, responsible for maintaining the appropriate partial pressure of O_2_ [2]. These endogenous factors determining the nodule functioning are strongly influenced by environmental cues, including the most dangerous abiotic factor for plants—drought. As we have previously shown, a soil water deficit reduces the number of formed nodules, leading to histological changes that indicate progressive symbiosome degradation; downregulates the expression of *LEGHEMOGLOBIN* (*LlLbI*); and decreases consequently the level of fixed N_2_ in *L. luteus* [3,4]. This is accompanied by a substantial accumulation of stress hormones—abscisic acid (ABA) and ethylene (ET)—in nodules [5]. At the same time, water deficit in soil induces a strong response in the aboveground parts of *L. luteus*, e.g., disrupting nutrient composition and enhancing flower abscission [5]. Flower separation processes in lupine involve the dissolution of middle lamellae formed between a specific group of cells, named the abscission zone (AZ) [6,7]. Similarly, as in the nodules, changes accompanying the flower abscission of *L. luteus* in response to drought are related to the modulation of biosynthesis pathways of stress hormones (ABA, ET), their accumulation, and distribution in AZ cells, as well as the induction of secondary stress mechanisms, which are reflected by a disruption of redox homeostasis [5,8]. Based on histological and immunofluorescent observations it can be hypothesized that drought modifies the cell wall structure in flower AZ [5,8]. These changes include the degree of homogalacturonans’ (HG) methyl esterification, upregulation of pectin methylesterase (PME), and polygalacturonase (PG), which catalyze subsequent reactions for pectin remodeling and disassembly in the middle lamella connecting adjacent cells [8].

The cell wall is a dynamic structure composed of a polysaccharide-based skeleton, proteins, and polymers organized in a complicated, dynamic network [9]. It represents the first cellular barrier and at the same time a defense line against stresses. The ability to modify the cell wall composition is one of the crucial factors which enables plants to adapt to and live under unfavorable conditions, such as drought. In *L. luteus*, cell wall changes taking place in the AZ determine the time of the organ detachment, while those that appeared in nodules enable the penetration of infection threads into the roots [10]. Given this important role of the cell wall in the regulation of both processes, here, we aimed to describe the modifications taking place simultaneously in nodules and the flower AZ of *L. luteus* growing under soil water deficit conditions. Understanding how the plant counteracts the negative effects of drought might be helpful for the improvement of the crop′s resistance to stress in the future.

Maintaining the cell wall integrity and proper architecture is crucial especially under the influence of different stress factors which may disturb the stability of plant cells, tissues, and whole organs. The primary cell wall is composed of cellulose microfibrils which are interconnected by polysaccharides—pectins and hemicelluloses [11]. Cellulose microfibrils provide the tensile strength of the cell wall and consist of long, unbranched chains of β-1,4-linked glucose units [9]. Pectins are branched molecules containing many negatively charged galacturonic acid units and form homogalacturonan (HG), xylogalacturonan (XGA), rhamnogalacturonan-I (RG-I), and RG-II [12]. Among pectins, HG contributes up to about ~65% of the total pool and can be modified by esterification processes, which ensures mechanical properties of the cell wall structure [13]. XGA is composed of HG substituted with a β-linked xylose [12]. Reports suggest that XGA may improve the resistance of HG to degradation by polygalacturonase (PG) acting under stressful conditions [14]. Other cell wall components, like RG-I, contain a motif of α-(1,4)-galacturonic acid and α-(1,2)-rhamnose and make up ~20–35% of pectin, whereas RGII consists of α-(1,4)-galacturonic acid only and represents ~10% of pectin [12]. RGI backbones could be linked to oligosaccharides, e.g., α-(l,5)-arabinans and β-(1,4)-galactans [15]. The middle lamellae connecting the cell walls of neighboring cells are mainly composed of pectins, which are therefore molecules fundamental to cell wall flexibility and cell-to-cell adhesion. Pectins can interact with arabinogalactan proteins (AGPs), in which protein moiety and carbohydrate polymers, e.g., D-galactose and L-arabinose, are present, and these compounds may stabilize the cell wall structure [16]. Among hemicellulosic components of the primary plant cell wall, the most common are xyloglucans (XyGs), which consist of a β-1,4-linked glucan backbone that is further linked to xylosylside chains. These chains can be in turn connected with galactose and fucose [17]. Interestingly, XyGs can be acetylated, and then their ability to link with other cell wall compounds is disrupted [18]. XyGs usually fill the spaces between microfibrils; however, in specific circumstances, the appearance of XyGs favors the formation of cellulose-xyloglucan-cellulose sandwiches, also named ‘biomechanical hotspots,′ which are exposed to the action of expansins–enzymes promoting the relaxation or extension of cell walls in a pH-dependent manner [17]. To summarize, given the ability of pectins and XyGs to bind to microfibrils, they can mediate both the generation of microfibrils cross-links, as well as their separation processes.

Proteins found in the cell wall make up about 5% of the wall′s dry mass; however, their role is invaluable, since most of them are enzymes playing roles in cell wall turnover and loosening [19]. These proteins are particularly important in the abscission processes because they modulate cell adhesion and ensure cell rupture, which is necessary for the separation to occur. In the scientific literature, there are reports regarding the identification of genes encoding enzymes responsible for the synthesis and modification of cell wall components during abscission in several plant species [20,21,22,23]. Considering these proteins, special attention should be given to extensins (EXTs), which belong to the family of plant cell wall hydroxyproline-rich glycoproteins (HRGPs). EXTs are also responsible for the mechanical properties of the wall, e.g., increasing strength [24]. Some studies have described EXTs as able to anchor polysaccharides in the cell wall and increase stress tolerance [25,26,27].

At present, cell wall components and their functions under water deficit conditions are poorly understood, particularly in crops such as lupines. Thus, in this paper, we focus on the drought-related localization of specific cell wall components including glycoproteins (extensins), pectins (HG, RG-I, XGAs), and hemicelluloses (XyG) which were monitored using monoclonal antibodies. Pectins were additionally measured using dot blot. Our analyses helped to identify new elements responsible for cell wall rearrangement in the nodules and flower AZ for the first time in lupine under soil drought. This is a piece of valuable information for modern agriculture and the selection of drought-resistant crops characterized by improved yielding. It is a base for the searching of elements that can modulate cell wall components and enzymes sensitive to drought and improve lupine tolerance to this stress factor.

## 2. Results

### 2.1. Verification of the Stress Conditions

**Figure 1:**
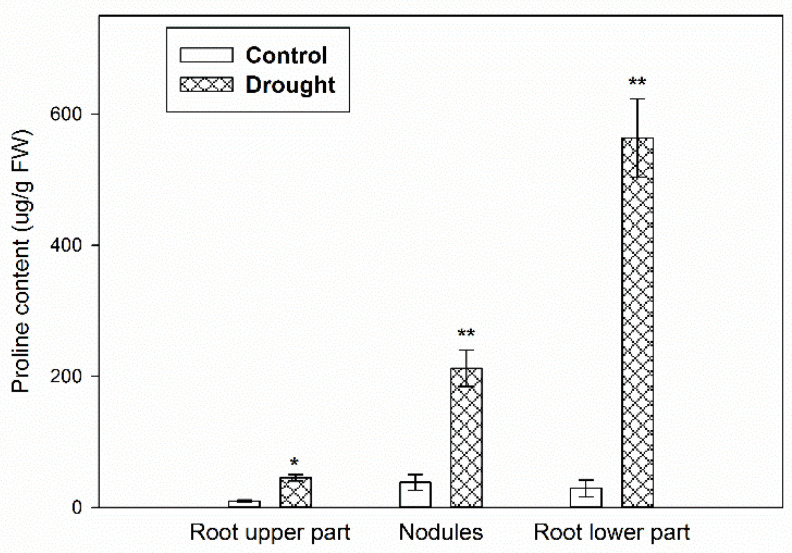
Proline accumulates differently in various parts of *Lupinus luteus* L. root subjected to soil drought. Control plants were grown under optimal conditions (70% water holding capacity, WHC), whereas stressed plants were subjected to water deficit conditions for 2 weeks (25% WHC). Samples were collected from different parts of roots and nodules on the 48th day of cultivation. Data are presented as averages ± SE. ** *p* < 0.01, * *p* < 0.05 (root part from stressed plant versus non-stressed ones) (Student’s *t*-test).

When we consider the influence of drought on the nodules and flower AZ functioning in *L. luteus*, the application of 25% WHC was effective, as we recently found. In such conditions, the number of nodules was reduced [5], and flower abortion was strongly stimulated [4]. This was correlated with the accumulation of proline—a drought stress marker—in the flower AZ [8]. In this paper, we show that water deficit in the soil leads to the accumulation of proline also in different parts of the root, including nodules (Figure 1), which were extremely affected by drought, as visible on microscopy images, especially in the fixation zone area (Supplementary Figure S1). The strongest stimulatory effect occurred in the lower part of roots, in which the level of proline was approx. 560 ug g^−1^ FW. In turn, the osmoprotectant content observed in nodules was 10 times higher under drought conditions when compared to control. 

**Figure 2:**
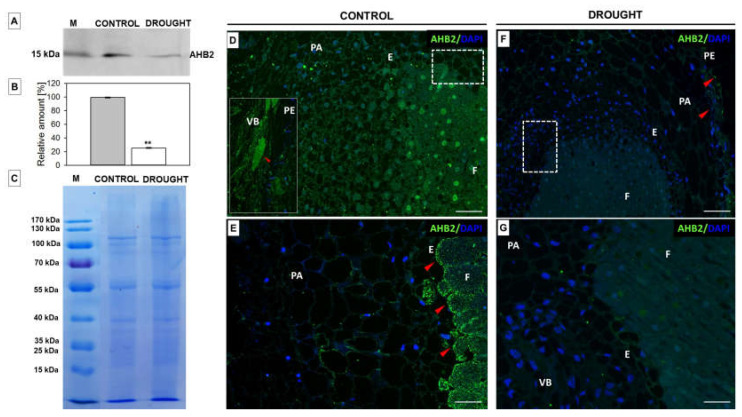
The level and localization of hemoglobin in nodules of *Lupinus luteus* L. are strongly affected by drought. Lupines were cultivated under water deficit conditions (25% water holding capacity, WHC), while control plants were grown in the soil of optimal moisture (70% WHC). For analysis, nodules were excised on the 48th day of cultivation. Western blot analysis was performed with AHB2 antibody (**A**). M–molecular mass marker. A band reactive to this antibody (of ~17 kDa) was scanned, densitometry analysis was made, and results are presented on (**B**) (100% was set for the control). Data are presented as averages ± SE. ** *p* < 0.01 (*n* = 3), Coomassie-Blue-stained gel as reference protein standard (**C**). The localization pattern of hemoglobin (AHB2 antibody) in stressed (**F**,**G**) and control (**D**,**E**) nodules. Images (**E**,**G**) are magnifications of regions of the fixation zone and parenchyma cells (white squares) from control and stressed nodules, respectively. Green fluorescence indicates AHB2 presence, whereas blue color corresponds to nuclei (DAPI staining). Red arrows denote strong fluorescent labeling. Abbreviations: F: fixation zone; E: endodermis; PA: nodule parenchyma; PE: periderm; VB: vascular bundle. Bars = 100 µm (**D**,**F**), 50 µm (**E**,**G**).

Given that leghemoglobin is crucial for the functioning of *L. luteus* nodules [3,4], here we aimed to verify the influence of soil drought on the level and localization of hemoglobin using the AHB2 antibody. Western blotting analysis revealed that the AHB2 (~17 kDa) level decreased following drought in root nodules (Figure 2A). The immunocytochemical analysis confirmed the presence of hemoglobin in control nodules (Figure 2D,E). Green fluorescence indicating hemoglobin localization was visible in the fixation zone (Figure 2E) and vascular bundle connecting nodules with the root (Figure 2D). Substantial differences in the AHB2 localization were observed when comparing the fixation zone and parenchyma cells (Figure 2E). On the contrary, almost no detectable fluorescence was visible in the drought-treated nodule (Figure 2F,G). Some fluorescent spots were detected in the external tissue surrounding nodules—the periderm (Figure 2F).

### 2.2. Drought Stress Affects Extensin Localization Pattern in Nodules and Floral Abscission Zone of Yellow Lupine

**Figure 3:**
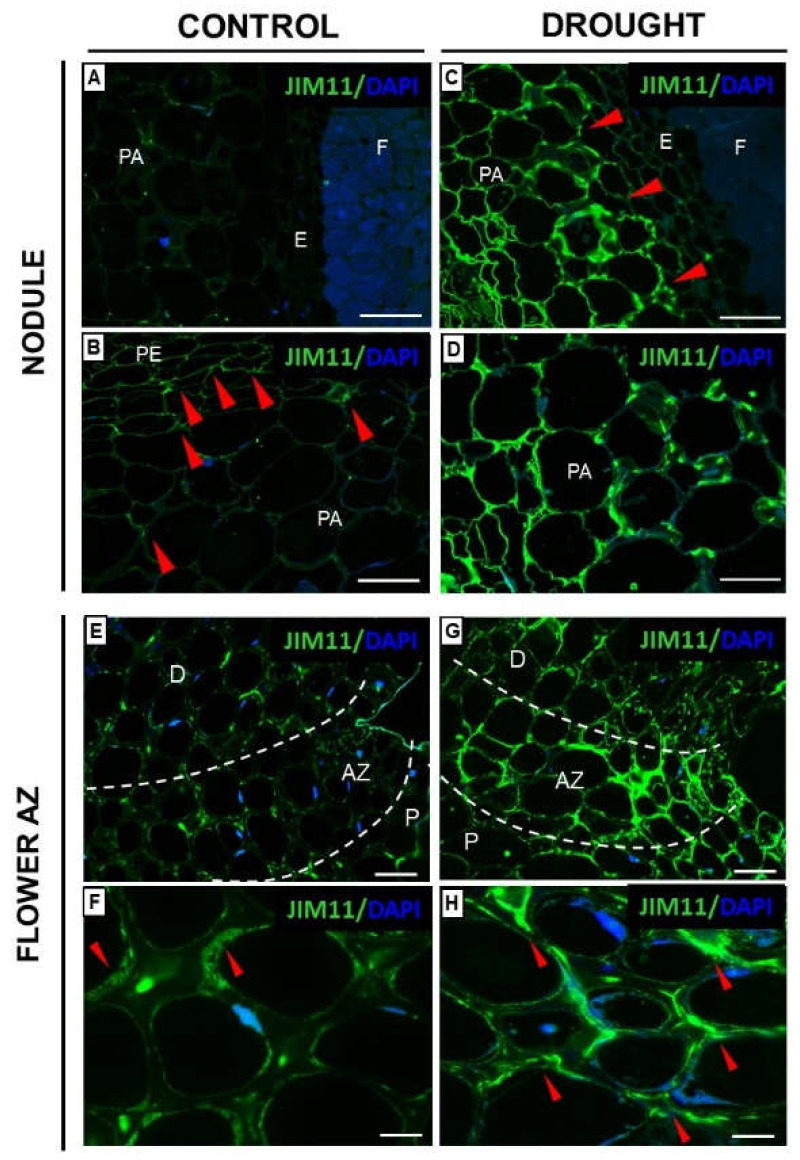
Drought stress changes the extensin (EXT) localization in nodules (**A**–**D**) and flower abscission zone (AZ) (**E**–**H**) of *Lupinus luteus* L. Immunolocalization of EXT (using JIM11 antibody) was performed in the nodules (**C**,**D**) and flower AZ (**G**,**H**) dissected from drought-treated plants (25% water holding capacity, WHC) and control lupines (70% WHC). Control nodules and AZ are presented on (**A**,**B**,**E**,**F**) respectively. For the analysis, tissue fragments were collected on the 48th day of plant cultivation. Images (**B**,**D**) are magnifications of parenchyma regions from control and stressed nodules, respectively. Images (**F**,**H**) show magnification of AZ region (white dotted lines) presented on (**E**,**G**). Green fluorescence indicates EXT detection, whereas DAPI blue labeling corresponds to nuclei. Red arrowheads indicate a strong signal. Abbreviations: F: fixation zone; E: endodermis cells; PA: nodule parenchyma; PE: periderm; AZ: abscission zone; P: proximal region of AZ, D: distal region of AZ. Bars = 50 µm (**A**,**C**), 25 µm (**B**,**D**), 40 µm (**E**,**G**), 20 µm (**F**,**H**).

**Figure 4:**
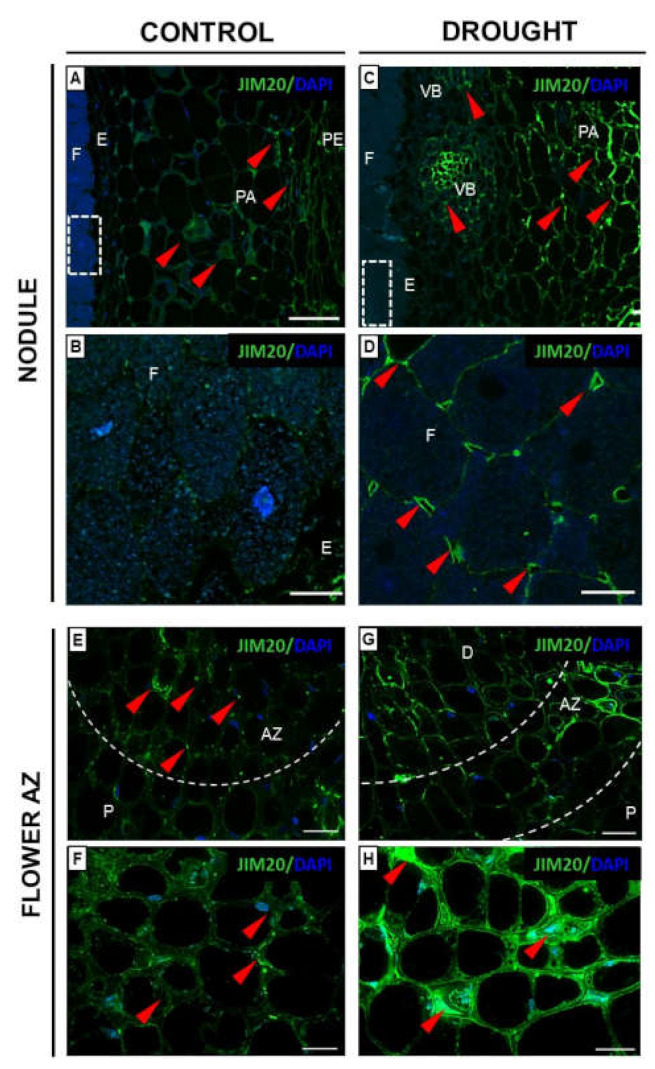
Extensin (EXT) distribution in nodules and flower abscission zone (AZ) cells of *L. luteus* L. is modified by soil drought stress. Immunolocalization of EXT (using JIM20 antibody) was performed in the nodules (**C**,**D**) and flower AZ (**G**,**H**) from drought-treated lupines (25% water holding capacity, WHC). Control nodules and AZs (70% WHC) are presented on (**A**,**B**,**E**,**F**) respectively. For each analysis fragments of nodules and AZs were collected on the 48th day of plant cultivation. Images B and D are magnified regions of the fixation zone from control and drought-treated nodules (squares on (**A**,**C**)). Images F and H show AZ region (white dotted lines) present in (**E**,**G**). EXT presence corresponds to green staining (red arrowheads), while blue fluorescence indicates nuclei (DAPI staining). Abbreviations: F: fixation zone; E: endodermis cells; PA: nodule parenchyma; PE: periderm; VB: vascular bundle; A: abscission zone; P: proximal region of AZ; D: distal region of AZ. Bars = 50 µm (**A**,**C**), 15 µm (**B**,**D**), 40 µm (**E**,**G**), 20 µm (**F**,**H**).

Extensins (EXTs) are glycoproteins that enable cell-wall assembly and growth by cell extension and expansion [28], e.g., during drought resistance, like in the case of wheat [29]. Therefore, we investigated the localization of these compounds under drought in nodules and flower AZ (Figure 3 and Figure 4). The results obtained by using two antibodies against EXTs indicate that soil drought stress caused the accumulation of these compounds both in nodules and the flower AZ of *L. luteus* (Figure 3 and Figure 4). A strong signal after reaction with JIM11 which identifies the isotype, IgG2c, was emitted by nodule parenchyma cells (Figure 3C,D), but was not noticed in the fixation zone (Figure 3C). Such strong labeling was not observed in the control nodule (Figure 3A,B). Some fluorescent spots were present in the parenchyma (Figure 3A,B), while in the periderm, a signal was emitted only by thin cell walls (Figure 3B). The fixation zone remained unlabeled both in the drought-treated nodule (Figure 3A), as well as in the case of control (Figure 3C).

Further study of the involvement of EXT revealed the presence of isotype IgG2c in the flower AZ of *L. luteus* (Figure 3E–H). Labeling intensity after reaction with the JIM11 antibody was weaker in the cell walls of AZ in the control plant (Figure 3E) than in the stressed ones (Figure 3G). Detailed analysis showed that the EXT in the control AZ was present in the cytoplasmic area (Figure 3F), while in the drought-treated AZ the signal was found mainly in the cell walls (Figure 3G).

Subsequent analyses have shown that the signal characteristic for EXT isotype IgM obtained after reaction with JIM20 was detected both in the nodules and flower AZ (Figure 4). Strong differences in the fluorescent labeling between drought-treated (Figure 4C) and control nodules (Figure 4A) have been observed. These EXTs were particularly detected in the vascular bundle and parenchyma cells of stressed nodules (Figure 4C); however, single luminous spots appeared in the area of periderm and parenchyma cells in the control sections (Figure 4A). The localization of the examined glycoproteins varied clearly in the fixation zone cells of both variants. Drought causes the accumulation of the EXT epitopes in the cell walls, particularly in the cellular junctions (Figure 4D), while the control fixation zone was practically free of labeling (Figure 4B). The fluorescent signal indicating the presence of this epitope of EXT formed a labeled area in the drought-treated AZ (Figure 4G). A quite different localization pattern, reflected by some fluorescent points, characterized the control AZ (Figure 4E). At higher magnifications, JIM20 immunofluorescence in the cell wall of the AZ was shown (Figure 4F,H); however, the intensity was higher in the case of stressed tissue in comparison to the control (Figure 4H).

### 2.3. Pectin Redistribution in Nodules and Flower Abscission Zone of Lupine Depends on Drought

**Figure 5:**
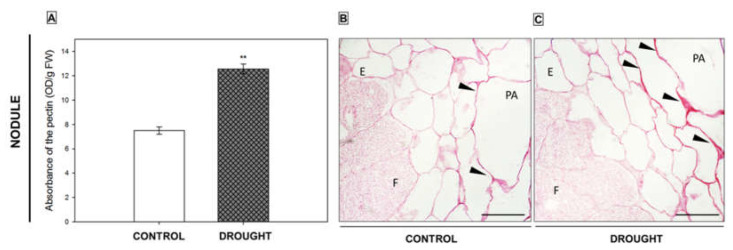
Drought affects pectin distribution and esterification degree in nodules of yellow lupine. Nodules were collected on the 48th day of cultivation from plants growing under soil drought (25% water holding capacity, WHC), and those grown in the soil of optimal moisture (70% WHC). The total pool of pectin, based on its absorbance, is presented on (**A**) (± SE, *n* = 3). Significant differences in the stressed plant in comparison to control are ** *p* < 0.01 (Student′s *t*-test). De-esterified pectin staining using ruthenium red in the control nodules (**B**) and drought-stressed ones (**C**). Black arrowheads are used to mark the strong pink staining reflecting the accumulation of de-esterified pectins (**B**,**C**). Abbreviations: F: fixation zone; E: endodermis cells; PA: nodule parenchyma. Bars = 50 µm (**B**,**C**).

As we have previously shown, soil drought stress caused the accumulation of pectin in the flower AZ of *L. luteus* [8]. We have also found that the activation of this structure upregulates PME and PG and leads to pectin de-esterification as a consequence, reflected by an increased level of demethylated homogalacturonan [8]. In this paper, we show that soil water deficit significantly increased the pectin level in the nodules too (Figure 5A) by almost 70% in comparison to control. We further aimed to verify whether the methylation level of these compounds changed in response to water stress. First, we performed staining with ruthenium red (Figure 5B,C). As Figure 5 shows, the cell wall of the parenchyma of drought-stressed nodules was stained more strongly (Figure 5C) when compared to control nodules (Figure 5B), which suggests an accumulation of de-esterified pectin. 

#### 2.3.1. Homogalacturonan

**Figure 6:**
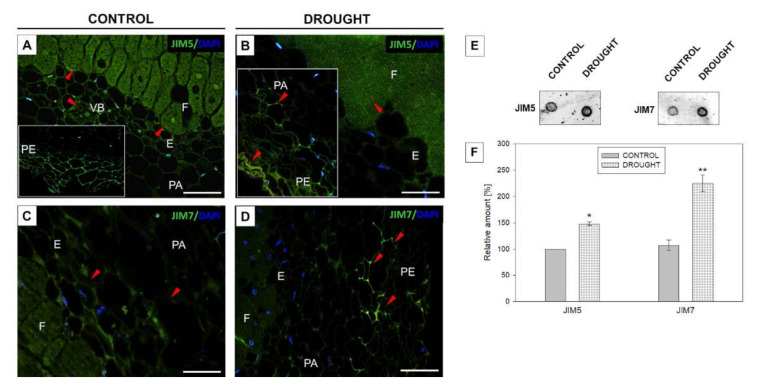
Drought has an impact on pectin methylation in lupine nodules. Low-methylated (JIM5 antibody) and high-methylated (JIM7 antibody) pectin was localized in the nodules of yellow lupine grown under drought conditions (**B**,**D**) and optimal soil moisture (**A**,**C**). Nodules were excised from roots on the 48th day of cultivation. The insert was put in A to highlight the signal emitted by the periderm area, while the insert in B shows fluorescence in the parenchyma and periderm region. Green labeling indicates methylated pectin presence (marked by arrowheads), while blue fluorescence corresponds to nuclei (DAPI staining). Abbreviations: F: fixation zone; E: endodermis cells; PA: nodule parenchyma; PE: periderm cells; VB: vascular bundle. Bars = 50 µm (**A**,**B**), 100 µm (**C**,**D**). Dot blot assay using the same primary antibodies as for the microscopy experiment (**E**). Analysis was performed on control and drought-treated nodules. Dots were scanned and normalized; densitometry values were quantified using ImageJ software (**F**). A value of 100% was set for the controls. Data are presented as averages ± SE (*n* = 3). Significant differences in stressed-nodules versus control are * *p* < 0.05 for JIM5 and ** *p* < 0.01 for JIM7 (*n* = 3).

Bearing in mind the results of ruthenium red staining, in the next step we performed comprehensive immunocytochemical analyzes to check the presence of pectins characterized by different methylation levels in nodules. We used monoclonal antibodies to detect low- (31–40%) and non-methylated HGs (JIM5 Ab) or high-methylated (15–80%) HGs (JIM7 Ab) [30]. Differential localization of both types of pectin in drought-treated and control nodules was found (Figure 6). Low-methylated pectin appeared in the control nodules; specifically in the cell wall of the periderm (Figure 6A, insert), cells that surrounded vascular bundles, and cells of the fixation zone (Figure 6A). When the drought was applied, a quite different localization pattern of de-esterified pectins was observed. These compounds were highly abundant in the periderm, while parenchyma remained unlabeled (Figure 6B). The periderm of stressed nodules (Figure 6D) and the endodermis of control (Figure 6C) were intensely stained after reaction with the JIM7 antibody. In the next step, we used the same antibodies (JIM5 and JIM7) for quantitative dot blot analysis (Figure 6E,F). The intense signal for both antibodies was higher in stressed nodules in comparison to control.

#### 2.3.2. Rhamnogalacturonan Type I

**Figure 7:**
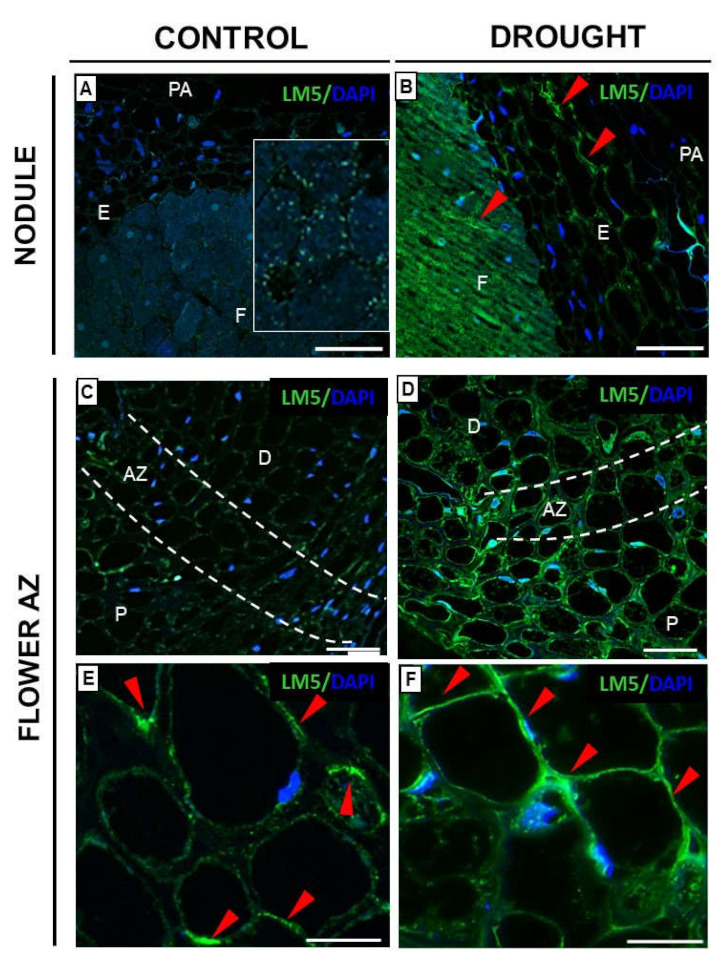
Galactans are accumulated in nodules and flower abscission zone (AZ) of yellow lupine following soil drought stress. Monoclonal antibody (LM5) was used to label (1-4)-β-D-galactans, RG–I side chain in the nodules (**B**) and AZ (**D**,**F**) of stressed plants (25% water holding capacity WHC), and also nodules (**A**) and AZ (**C**,**E**) of control lupines cultivated in optimal moisture (75% WHC). Insert is a magnified region of the fixation zone presented on (**A**). For immunofluorescence analyses, tissues were excised on the 48th day of cultivation. Green fluorescence indicates RG-I localization (red arrowheads), while a blue signal is emitted by nuclei (DAPI staining). AZ area is marked by white dotted lines on (**C**,**D**) and magnified on images (**E**,**F**). Abbreviations: **F**: fixation zone; E: endodermis; PA: nodule parenchyma; AZ: abscission zone; P: proximal region of AZ, D: distal region of AZ. Bars = 50 µm (**A**,**B**), 60 µm (**C**,**D**), 25 µm (**E**,**F**).

**Figure 8:**
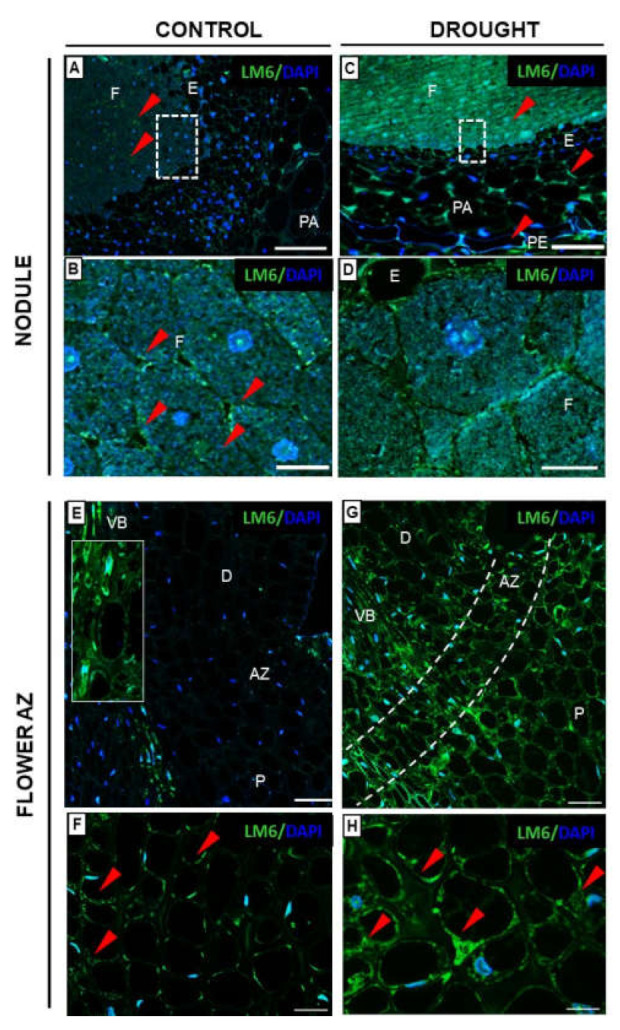
**Drought affects the localization of arabinan in nodules and flower AZ of lupine.** Monoclonal antibody LM6 served to detect (1-5)-α-L-arabinans RG-I side chains in the nodules (**C**,**D**) and flower AZs (**G**,**H**) of plants cultivated under drought conditions (25% water holding capacity, WHC). Nodules (**A**,**B**) and AZs (**E**,**F**) from lupines growing in optimal moisture (75% WHC) were the control. Material for analysis was collected from 48-day-old lupines. Green fluorescence indicates arabinan presence (red arrowheads), whereas DAPI blue signal is emitted by nuclei. Images (**B**,**D**) are magnifications of fixation zone areas from (**A**,**C**) (white squares). White dotted lines on (**E**,**G**) correspond to the AZ area, which is magnified precisely on (**F**,**H**), respectively. Additionally, a small square on E shows an enlarged region of vascular tissue from the pedicel. Abbreviations: F: fixation zone; E: endodermis; PA: nodule parenchyma; PE: periderm; VB: vascular bundle; AZ: abscission zone; P: proximal region of AZ, D: distal region of AZ. Bars = 50 µm (**A**,**C**), 15 µm (**B**,**D**), 100 µm (**E**,**G**), 20 µm (**F**,**H**).

The flexibility and degree of stiffening of the cell walls in response to stress depend on the content and/or mobility of galactans and arabinans, which belong to a group of RGI [12]. Here, we investigated the tissue localization pattern of both types of pectins in nodules and flower AZ of plants subjected to water stress (Figure 7 and Figure 8). A signal indicating the presence of galactans detected by the LM5 antibody was observed in the fixation zone and endodermis of drought-treated nodules (Figure 7B). In control, single, luminous points were detected only in the cell wall of the fixation zone, with the remaining tissues unlabeled (Figure 7A, insert). 

Furthermore, galactans were detected also in the drought-activated AZ of flowers (Figure 7D). The fluorescence spread to the distal and proximal region of this structure. Such a strong effect was not observed in the control AZ (Figure 7C). At higher magnification, green labeling was visible in the peripheral regions of the cytosol of control AZ (Figure 7E). Importantly, much stronger fluorescence in the cytosol and cell walls of drought-stressed tissue was observed after reaction with the LM5 antibody (Figure 7F).

#### 2.3.3. Xylogalacturonan (XGA) Localization Is Regulated by Drought in Nodules and Flower AZ

**Figure 9:**
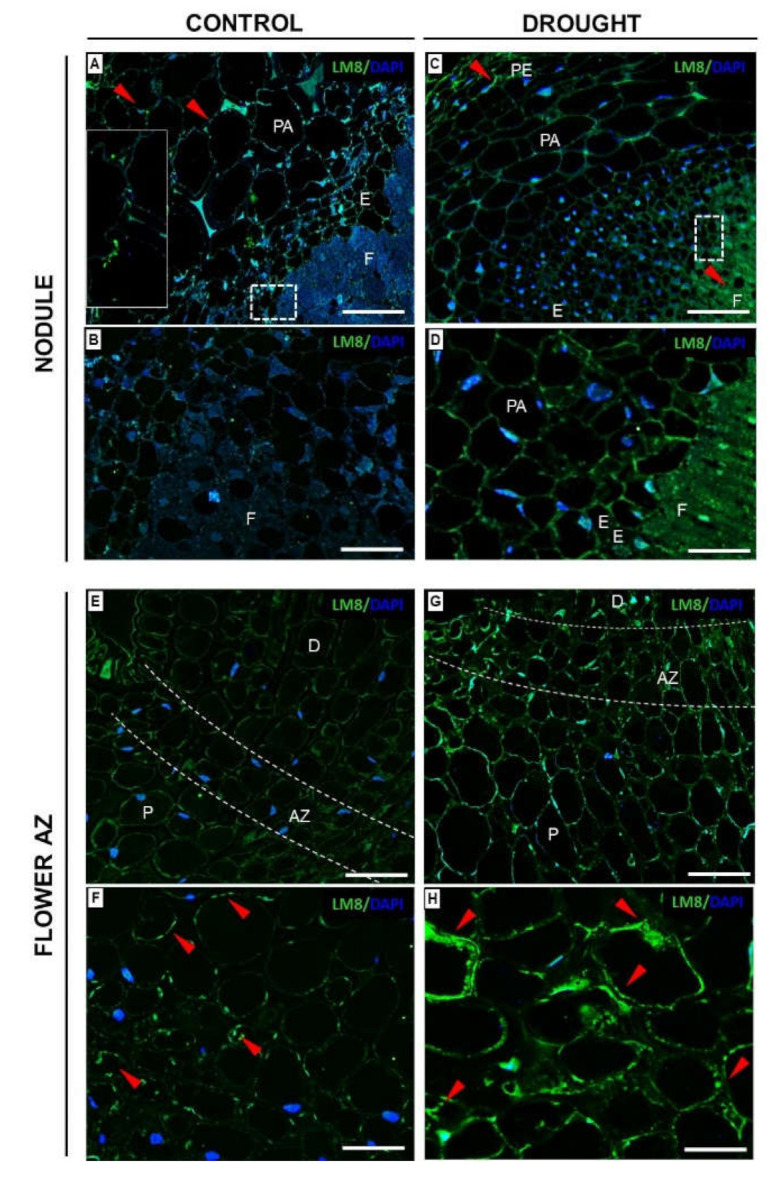
Soil drought stress changes xylogalacturonan (XGA) localization in the nodules and flower abscission zone (AZ) of yellow lupine. Monoclonal antibody (LM8) was used to label XGA in the nodules (**C**,**D**) and AZ (**G**,**H**) of stressed plants (25% water holding capacity, WHC). Reactions were made also for control nodules (**A**,**B**) and AZ (**E**,**F**) from plants cultivated under optimal moisture (75% WHC). Material for analysis was harvested on the 48th day of lupine cultivation. Green fluorescence corresponds to XGA localization, while blue labeling is visible in the presence of nuclei after DAPI staining. (**B**,**D**) are magnifications of regions of fixation zones. Left insert on (**A**) shows the parenchyma region. Enlarged regions of AZ (marked by white dotted lines on (**E**,**G**) are presented on (**F**,**H**). Red arrowheads indicate a strong signal emitted from different compartments. Abbreviations: F: fixation zone; E: endodermis; PA: nodule parenchyma; PE: periderm; VB: vascular bundle; P: proximal region of AZ, D: distal region of AZ. Bars = 50 µm (**A**,**C**), 25 µm (**B**,**D**), 80 µm (**E**,**G**), 40 µm (**F**,**H**).

We checked the distribution of XGA in the nodules and flower AZs from lupines subjected to drought. In stressed nodules, the signal for XGA antibody was emitted by the fixation zone (Figure 9C,D) and periderm cells (Figure 9C). In turn, for control nodules, fluorescence was noticed in the parenchyma (Figure 9A, insert), while the fixation zone remained unlabeled (Figure 9B). The presence of the tested polysaccharide in the AZ was not restricted to AZ cells. It was detected also in the distal and proximal parts of both examined variants-control and drought (Figure 9E,G). A high green signal was observed in the cytosolic region and intracellular spaces of stressed AZ (Figure 9H). As Figure 9 shows, the LM8 labeling was much weaker in AZ cells from control plants (Figure 9F).

### 2.4. Modification in Hemicellulose Composition in AZ cells under Drought Is Reflected by Changes in Xyloglucan Localization

**Figure 10:**
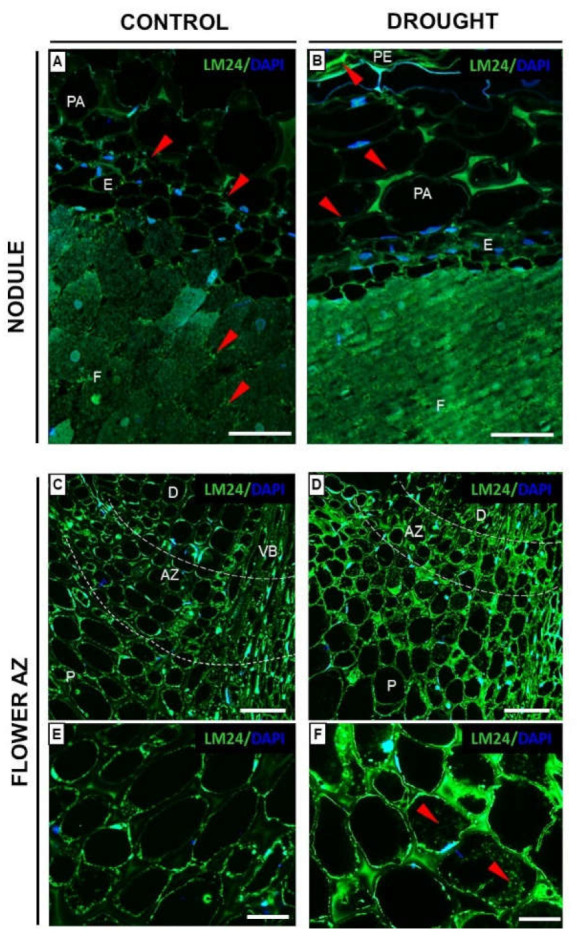
Nodule- and flower-AZ-specific distribution of xyloglucan (XyG) in yellow lupine subjected to drought. LM24 antibody was used to detect XyG in nodules (**B**) and AZ (**D**,**F**) collected on the 48th day of lupine cultivation under drought stress (25% water holding capacity, WHC). Control sections were prepared from nodules (**A**) and AZ (**C**,**E**) of plants growing in the soil of optimal moisture (75% WHC). Green and DAPI-blue fluorescence indicate XyG and nucleic acid staining, respectively. AZ region is limited by white dotted lines (**C**,**D**). Enlarged regions of AZ are on images (**E**,**F**). Red arrowheads indicate the areas characterized by strong fluorescence. Abbreviations: F: fixation zone; E: endodermis; PA: nodule parenchyma; PE: periderm; VB: vascular bundle; AZ: abscission zone; P: proximal region of AZ, D: distal region of AZ. Bars = 30 µm (**A**,**B**), 100 µm (**C**,**D**), 25 µm (**E**,**F**).

Xyloglucan (XyG) is the most abundant hemicellulose in plants [17]. Here, we performed the immunocytochemical analysis with the LM24 antibody detecting the XLLG motif of XyG in the cell walls (Figure 10). The water deficit in the soil caused the accumulation of XyG in all tissues of the nodules—the fixation zone, endodermis, parenchyma, and periderm (Figure 10B). A strong signal was emitted especially by the cell wall of the parenchyma and periderm region (Figure 10B). Much weaker fluorescence was observed in the sections made from the control nodule (Figure 10A). When we analyzed the flower AZ, we noticed that the whole region is labeled, both in control (Figure 10C) and stressed tissues (Figure 10D). At higher magnification, XyG occurs in the cell walls (Figure 10E,F). Furthermore, clusters emitted fluorescence from the cytoplasm of drought-treated AZ cells (Figure 10F). Lower xyloglucan content was visible in the flower AZ of plants growing under optimal moisture conditions (Figure 10E).

### 2.5. Polysaccharides Are Strongly Accumulated in Nodules and Flower AZ of Yellow Lupine under Drought Conditions

**Figure 11:**
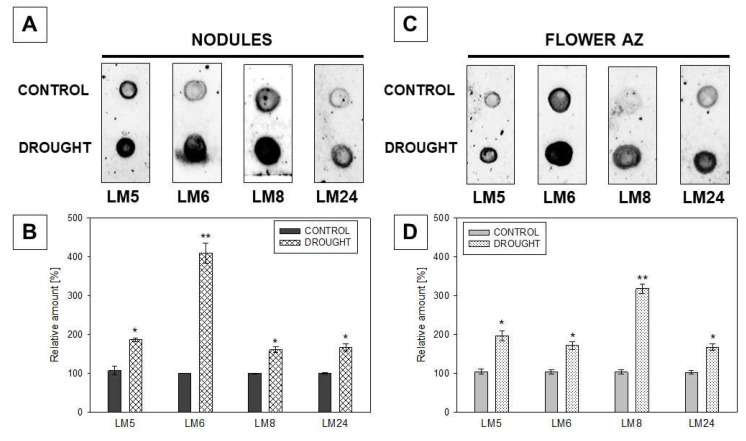
Pectin antigens are differentially present in the drought-treated nodules (**A**) and flower abscission zone (AZ) (**C**) of lupine. Immuno-dot-blot by using LM5, LM6, LM8, and LM24 antibodies was applied to detect galactans, arabinans, xylogalacturonans, and xyloglucans, respectively. The same primary antibodies as for the microscopy experiment were used. Tissues were collected on the 48th day of lupine cultivation under drought stress (25% water holding capacity, WHC) or plants growing in the soil of optimal moisture (75% WHC, control). The same amount of proteins (2.5 µg) was loaded for each dot. Dots were scanned and normalized densitometry values were quantified using ImageJ software for nodules (**B**) and AZ (**D**) tissues. A value of 100% was set for the controls. Data are presented as averages ± SE (*n* = 3). Significant differences in stressed tissues versus control for each antibody are * *p* < 0.05 and ** *p* < 0.01 (*n* = 3).

These immunofluorescent observations prompted us to more precisely analyze the influence of drought on the polysaccharide content in both types of tissues. We applied the immuno-dot-blot test using LM5, LM6, LM8, and LM24 antibodies (Figure 11). Despite substantial variations, the signal for all antibodies in drought samples was much stronger when compared to non-stressed control, both for nodules (Figure 11A,B) and flower AZs (Figure 11C,D). Nevertheless, it seems that the presence of a particular polysaccharide is tissue-specific. For instance, in the case of nodules, the strongest signal was emitted after reaction with LM8 Ab recognizing XGA, but it did not show such reactivity for AZ. Considering treatment with drought, the antigens were accumulated more in nodules than in AZ, apart from LM6, which binds to arabinose-rich pectins.

### 2.6. Fixation Zone-Specific Arabinogalactan Presence Is Disrupted in Nodules under Drought 

**Figure 12:**
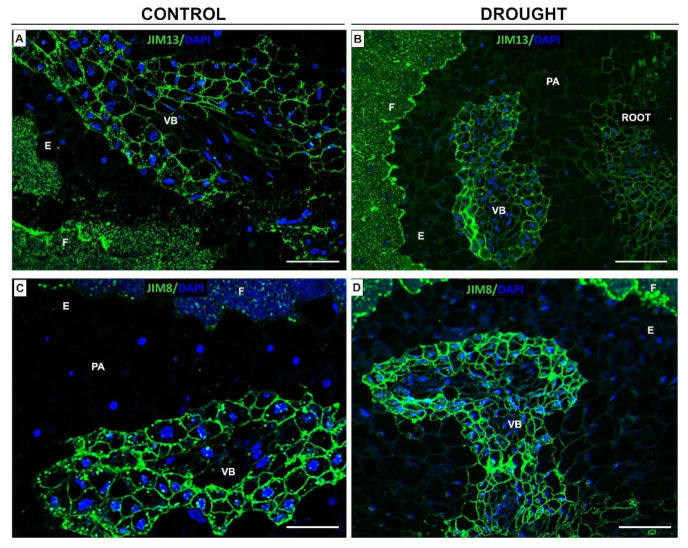
Water deficit stress affects arabinogalactan protein (AGP) localization in yellow lupine nodules. Immunofluorescence localization of different AGPs epitopes was made using JIM13 (**A**,**B**) and JIM8 (**C**,**D**) antibodies. Reactions were made for the control nodules (**A**,**C**) and those excised from plants growing under drought conditions (**B**,**D**). Tissues were collected from 48-day-old lupines. AGP presence corresponds to green fluorescence, while blue labeling indicates nuclei stained with DAPI. Abbreviations: F: fixation zone; E: endodermis cells; PA: nodule parenchyma; VB: vascular bundle. Bars = 75 µm.

Therefore, in the next step, we checked the impact of drought on the localization of arabinogalactan proteins (AGPs) in the structures responsible for bacteria–lupine interactions—nodules. We used JIM13 [31] and JIM8 [32] antibodies (Figure 12). It seems that JIM13 binds more effectively than the other tested antibody since the fluorescent signal is higher (Figure 12A,B). When we observed tissues after reaction with JIM8, the labeling was characterized for the vascular bundle of both stressed and control nodules (Figure 12C,D). In turn, the fixation zone was labeled strongly in drought-treated nodules when we compared it to the control (Figure 12C,D). A similar relationship was noticed when we used the JIM13 antibody; however, the labeling was even stronger (Figure 12A,B).

## 3. Discussion

Plants have evolved multiple strategies to counteract the negative impact of drought, such as molecular, biochemical, morphological, and physiological modifications which enable them to minimize the effects of stress and maintain proper homeostasis [33,34]. Knowledge about this phenomenon should be understood especially in economically important species, among them *Lupinus luteus*. The negative correlation between the yield of lupine and drought is a good reason to study the mechanisms triggered by this stress. Although nodules are important drought sensors, their response to water deficit is still poorly understood. At present, we have shown that the treatment of yellow lupine with a two-week drought in the stage of formation of generative organs, when plants have an increased demand for nutrients and water, causes dysfunction of the root nodules [5] and activation of AZ in flowers, leading to their separation [5,8]. As a consequence, the yielding of lupine is strongly affected. Given the negative impact of water deficit on nodulation and flower maintaining, it is crucial to examine the changes evoked by this stress both in nodules and the flower AZ.

Modifications characteristic for drought action were described in our previous analyses performed on yellow lupine nodules [4] (and in the present Supplementary Figure S1), and the flower AZ [5] and have been confirmed by the increasing level of an osmoprotectant—proline—both in the nodules (Figure 1) and floral AZ [8]. In the root, the highest level of proline was noted in the lower part, in which root hairs responsible for water uptake are formed. Such synthesis and accumulation of proline in the cytoplasm minimizes the damage caused by water deficiency [35].

Drought stress disrupts not only the structure of nodules [4] but also leghemoglobin activity [3]. Our results show a decrease in the level of hemoglobin in lupine nodules under drought stress, which supports the localization of this protein (Figure 2) after using a heterologous antibody to non-symbiotic AHB2, with a clear reactivity in lupine. Generally, in plants, two hemoglobin groups have been identified: symbiotic and non-symbiotic. The first of them are present in the nodules of *Fabaceae* plants [36]. These hemoglobins are involved in N_2_ fixation; however, they are not required for general growth developmental processes [37]. In turn, non-symbiotic hemoglobins are present in various tissues of many plant species, especially crops [38,39]. Non-symbiotic hemoglobins are divided into two classes: Hb1 and Hb2; Hb1 is characterized by high affinity to O_2_ and is involved in the hypoxia response [40], while the Hb2 role has not yet been described. It is only known that Hb2 participates in the response to cold stress and is associated with the functioning of young, active tissues with high energy requirements [41,42]. Research conducted on *A. thaliana* shows that AHB2 has a comparable affinity for O_2_ as leghemoglobin; therefore, its role in facilitating oxygen diffusion cannot be ruled out [43]. As shown here, the presence of non-symbiotic AHB2 hemoglobin in the plant tissue, as well as in the nodules of lupine under optimal water conditions, might suggest that this hemoglobin is involved in the proper functioning of the nodule, which requires a high amount of energy for metabolic activity. Such hypothesis can be supported by the strong signal emitted by the control fixation zone (Figure 2) and the decrease in N_2_ fixation in drought-stressed nodules that was observed previously [4].

The cell wall capacity of being stretched allows the plant cell to maintain turgor pressure, which is seriously disrupted under water stress conditions [44]. Thus, the cell wall strengthens the plant body and plays a key role in the growth processes, cellular differentiation, and communication. Cell wall components, e.g., extensins, pectins such as homogalacturonans (low- and high-methylated), rhamnogalacturonan (galactan and arabinan), xylogalacturonan, hemicelluloses (xyloglucan), and arabinogalactans are involved in these processes [12].

Generally, EXTs play a role in biotic and abiotic stress response, e.g., by pathogen attack, during which EXTs provide mechanical protection against pathogen invasion [45]; heavy metal ions; or mechanical wounding [46,47,48,49]. Furthermore, EXTs as glycoproteins enriched in arabinose might help to prevent the drying-out of tissues when water potential in cells is reduced [50]. Thus, immunocytochemical analyses of drought-resistant plants—*Myrothamnus flabellifolia*, *Craterostigma plantagineum*, *Xerophyta viscosa*, *Xerophyta schlecterii*, *Xerophyta humilis,* and *Eragrostis nindensis*—have revealed that their cell wall is enriched in pectins and EXTs [51]. Given these facts, we aimed to precisely localize EXTs under drought stress in nodules of *L. luteus* and following flower abscission using JIM11 and JIM20 antibodies. Castilleux et al. [46] proposed the epitope structure recognized by the JIM11 and JIM20 mAbs based on immunocytochemical and immunoblotting analyses of *A. thaliana* mutants disabled in EXT arabinosylation and wild-type roots. According to their observations, the JIM11 epitopes would contain the third arabinose and/or following arabinose residues in the glycan moiety of EXT, while the JIM20 epitope may include part or even the entire structure of the three first arabinoses and galactoses from the glycan moiety of EXT. Here, we found that water deficit induces a strong increase of JIM11 (Figure 3) and JIM20 (Figure 4) labeling in nodules, with differential detection, in the parenchyma cell wall and vascular bundles, respectively. Such distribution in stressed lupine is quite different than in other species under various stress, e.g., biotic, as described in the available literature. Thus, the defense reaction of *Benincasa hispida* to *Fusarium* caused EXT accumulation (detected by JIM11) in the cortex, rhizodermis, endodermis, and phloem, while JIM20 epitopes were present in the cortex, endodermis, phloem, and vascular parenchyma [52]. EXTs localized in parenchyma cells of *L. luteus* nodules under drought might play a role as a diffusion barrier, helping to protect the N_2_ fixing enzymatic apparatus against inactivation [53,54]. The different localization of both EXTs in the flower AZ of *L. luteus* (Figure 3 and Figure 4) could be related to their distinct role in stress response in this tissue. JIM11 recognized the EXT isotype present in the cell wall of drought-treated AZ (Figure 3), while the EXTs recognized by JIM20 localized both in the cell wall and cytosolic regions (Figure 4). Given the obtained results, we supposed that EXT present in drought-treated AZ is on the one hand responsible for keeping water in cells under drought, but on the other hand, EXTs could be involved in the cell wall remodeling processes required for AZ activation and organ abscission, both stretching and stiffening [55].

One of the adaptive features of plants living in stressful conditions is the ability to change the degree of pectin methylation in the cell wall [56,57]. The higher the esterification, the lower interaction of pectins with Ca^2+^ to form gels. Due to the presence of Ca^2+^, de-esterified pectins form a cross-link that stabilizes and mechanically strengthens the cell wall [58]. Changes in pectin methylation under drought have been observed in the roots of *Pisum sativum*, *Medicago truncatula*, and *Beta vulgaris*, and the leaves of *Zea mays* and *Populus* L. cambium [59,60,61,62,63].

Intensive pectin reorganization in response to drought has been observed previously in the flower AZ of *L. luteus* [8]. Here, we argue that a deficit of water in the soil leads to the accumulation of pectins in nodules (Figure 5) and that a change in their methylation degree also occurs (Figure 6). The stressor upregulates the low- and high-methylated pectins, which are differentially localized in nodule tissues (Figure 6). A similar tendency has been demonstrated in *Craterostigma wilmsii* [64]. In lupine, low-esterified pectins are observed in the parenchyma and periderm of nodules, whereas highly-esterified ones are detected mainly in the periderm (Figure 6). We may speculate that the degradative processes triggered by stress may involve cell wall destruction starting at the external part of nodule tissues—the periderm. In this regard, pectin might be involved in the thickening and stiffness of cell walls, thus promoting the formation of a protective layer [65,66]. Changing localization patterns of both high- and low-methylated pectin suggests that drought stress is accompanied by a modification of the methyl-esterification degree in different parts of the nodules, which could modify both the mechanical properties of their cell wall, their plasticity, and the possibility of infection by bacteria. We also show here that in lupine, the response of the cell wall of nodules and flower AZ to drought is reflected by accumulation (Figure 11) and the changing localization of galactans (Figure 7) and arabinans (Figure 8). Generally, arabinans are present mostly in young cells, while galactans have been demonstrated to be abundant in older ones. However, our observations, as well as those made on *P. sativum* and *M. truncatula*, indicate that this relationship could be species-specific [61]. In yellow lupine, the signals for both epitopes (arabinans and galactans) in mature control nodules are weak and are restricted to the cell wall of the fixation zone area (Figure 7 and Figure 8). In *P. sativum* and *M. truncatula* galactans labeled with LM5 were detected in the meristematic cells of nodules, while in the mature structures, the signal was emitted only by the endodermis and the vascular bundles [61]. Drought in lupine caused an accumulation of galactans and arabinans in the whole region of the nodule (Figure 7 and Figure 8). Both molecules are very mobile and flexible under changing hydration of cells [67]. Indeed, it has been shown that arabinans can be involved in cell rehydration after drought in potato [68]. Plants that are tolerant to desiccation have been described to be rich in arabinan [51]. Thus, the remodeling of cell walls involving the appearance of galactan and arabinan in lupine nodules under drought might be related to rehydration. Furthermore, a proportion of galactose and arabinose residues are also important for the structure and functions of EXT [28], which are accumulated in nodules under drought (Figure 3 and Figure 4). Considering the presence of galactan and arabinan in the AZ of different species, species-specific changes have been described. Thus, both are accumulated in the flower AZ of tomato following abscission [69]. The opposite relationship occurs in *Euphorbia pulcherrima* (poinsettia), in which the content of galactans and arabinans decreases under flower abscission, possibly due to the upregulated activity of hydrolytic enzymes such as galactanases and arabinases [70]. Interestingly, an accumulation of galactans and arabinans was demonstrated in the AZ of citrus fruit when a precursor of ethylene was applied [71]. We have also proven a strong association of drought-related events with ethylene, directly in the flower AZ [5]. Thus, ethylene is likely responsible for the accumulation of arabinans and galactans in response to drought. In our present study, galactans were specifically located in the cell walls (Figure 3), while arabinans were present either in the cell wall or inside the cell (Figure 4). We believe that these interesting observations are related to the synthesis of the components that build the cell walls formed between cells after divisions, which are characteristic for flower AZ activation in lupine [5,72]. Arabinans are possibly involved in the regulation of the distance between the HG chains, and in this way, they prevent HG interactions with Ca^2+^ [73]. The changing turgor of the cell, induced by stresses like drought, results in the high mobility of arabinans and galactans, which can fill free spaces formed by the rearrangement of other components. Arabinan has been suggested to ensure the flexibility of the structure of the cell wall during changes of cell volume and shape in *A. thaliana*, *Zea mays*, and *Phaseolus vulgaris* roots infected by a cyst nematode [74]. In turn, *Solanum tuberosum* mutants with a reduced content of arabinans and galactans are characterized by the lower elasticity of cell walls and their stiffness [75]. In this context, the accumulation of both arabinans and galactans reported here may indicate that the appearance of these compounds is induced by a water deficit reaction. In line with these findings is also the statement that synthesized arabinans and galactans can maintain cell integrity while protecting plant tissues shortly after the organ is abscised. However, it is not excluded that both kinds of molecules might interact with other cell wall components. Arabinan chains mediate the binding of cellulose, which provides mechanical force [76].

The changing level of XGA contributes strongly to the reorganization of plant cell wall structure [14], which could accompany drought-related events. The LM8 antibody recognizes XGA, which has an HG backbone with xylose residues linked by *β*-(1–3) bonds to galacturonic acid [77]. In yellow lupine, drought leads to the accumulation of LM8-epitope-containing XGA in the external part of nodules (Figure 9). Research conducted on *P. sativum* shows that the synthesis of XGA in the outer layers of root cells and nodule cortex accompanied drought [59]. The XGA appearing in the periderm of nodules could constitute the first protective barrier and thus may be an element of the mechanism triggered in response to drought stress.

There are several studies reporting modification of pectin composition following organ abscission processes taking place in the AZ cells of different species [69,70], and also in lupine [8]. However, only Bowling et al. [78] paid attention to the specific form of pectin–XGA contribution in abscission-related processes in oil palm. Here, we used LM8 antibody to check the localization of XGA in the flower AZ of lupine in response to drought (Figure 9). In contrast to the results of Roongsattham et al. [79] who noted the presence of XGA only after AZ cell separation, we observed upregulation of XGA in AZ cells when AZ is already induced to abscission (Figure 9). Xylose from XGA could prevent the Ca^2+^-dependent interactions between HG; however, the precise mechanism of its action is still unknown [13]. This scenario is possible in drought-treated lupine AZ, given that we used the LM8 Ab, which binds to an epitope of XGA possibly substituted with a xylose [30]. Further support comes from recent analyses by us, showing that drought modifies the methyl-esterification of HG controlled by PME in AZ of lupine flowers [8].

The main hemicellulose in dicots is XyG, which interacts with celluloses via hydrogen bonds [19,20]. Furthermore, XyG is secreted by root cells into the surrounding soil, where it acts as an efficient molecule in the formation of soil aggregates [80]. Nodulation requires the hydrolytic activity of nodulation outer proteins (NopAA), which help to indicate safe nodulation and are responsible for maintaining a low level of XyG, adequate enough for the initiation of infection. This could explain the low content of this molecule in nodules under normal, control conditions (Figure 10). The strong signal related to XyG appearance in the fixation zone of stressed nodules of *L. luteus* could represent evidence for the disturbed plant–pathogen interaction. Furthermore, the accumulation of XyG in parenchyma and periderm cells could be linked to cell wall stiffening. For instance, during heat stress the large amount of XyG could be hydrolyzed by xyloglucan endotransglucosylase/hydrolase (XTH), consequently causing a strengthening of cell walls, which is helpful in the adaptation to stress [81].

Drought also causes the accumulation of XyG in the cell walls of the whole flower AZ area (Figure 6). According to the literature, xyloglucans could be localized in the cell wall matrix [82]. They are strongly accumulated in the induced AZ of tomato and poinsettia flowers; however, during tomato fruit abscission, no AZ-specific cell wall polysaccharide deposition was detected [69,70]. Authors have proposed that xyloglucans can be substrates for cell wall degrading enzymes and/or act as protective substances that appear in response to AZ activation [69,70]. Transcripts corresponding to XTH, xyloglucan galactosyltransferase, xylogalacturonan beta-1.3-xylosyltransferase, glucan endo-1,3-beta-glucosidase, and expansins (EXPs) are accumulated during the abscission of rosa flower petals, *Sambucus nigra* leaves, and lupine flowers [83,84]. In the cell wall, cellulose microfibrils are linked by XyG, and xyloglucan endotransglucosylase (XET) rearranges their structures in a transglycosylation reaction, based on the hydrolytic cleavage of a glycosidic bond within one XyG molecule, and then transferring and joining its end to another fragment. The expansin located in the cell walls interrupts the formation of non-covalent bonds between cellulose microfibrils, which results in loosening the cell wall structure enabling its remodeling and cell enlargement [85]. There are also data confirming the possibility of HG binding to XyG via RG-I, including arabinans and galactans [86,87,88]. To sum up, the observed changes in XyG localization in the flower AZ of lupine under drought suggest that these compounds, possibly synthesized de novo, could be built in the polysaccharide cell wall matrix and/or might be exposed to the action of hydrolytic enzymes, e.g., XET or EXP.

The AGPs are a family of hydroxyproline-rich glycoproteins involved, e.g., in the response to abiotic and biotic stress factors [89], root growth and differentiation [90,91,92], and interactions with microorganisms [93,94,95]. During symbiotic host/microbe contacts, AGPs seem to interact with plant membrane via glycosylphosphatidylinositol [96]. The localization pattern of JIM13 and JIM8 observed here indicates that these epitopes may appear in different ways in lupine in response to drought (Figure 12). The appearance of JIM13 in the fixation zone of the *L. luteus* nodule might indicate its involvement in the regulation of symbiosis, similarly to the AGPs detected by JIM13 in *Alnus*, which were localized following early nodulation stages of nitrogen-fixing actinobacteria and after the formation of the infection thread [97]. This epitope is seemed not to be sensitive to drought in lupine, since the localization with JIM13 is unchanged in the fixation zone area after stress (Figure 12). Contrarily, the different localization patterns of the JIM8 epitope in drought-stressed and control nodules of lupine (Figure 12) could indicate that these AGPs take part in the reorganization of the cell wall in response to drought, which helps to protect roots against stress. Generally, AGPs are localized in different tissues, including developing vascular bundles and neighboring cells [98,99], which might suggest that they are involved in the regulation of plant growth, development, and architecture. Thus, the appearance of AGPs in the vascular system in yellow lupine root nodules could be related to their function in the formation and morphogenesis of the vascular system in the root area. The precise mechanism of this function is not yet known, although evidence suggests that AGPs are involved in cell elongation and growth [100] since they affect the organization of microtubules [101].

In the presented paper, we determined for the first time in lupine the drought-associated modifications of cell wall structure including cell wall compounds. Moreover, we have identified proteins or protein families responsible for such cell wall remodeling in root nodules and flower AZ of lupine. Cellular analyses presented here help to identify the precise tissues involved in the modifications, and also suggest a number of potential actors (genes or pathways) for this remodeling, as a basis for further biochemical and genetic complementary analyses. Future perspective could include, e.g., the use of gene editing or mutants characterized by delayed/overexpression of specific genes like those involved in proline synthesis, leghemoglobin, PME, and expansins, etc., as well as QTL or GWAS analyses. Comparative transcriptomic and proteomic approaches have also demonstrated their use to advance drought stress understanding [102,103,104]. These “-omic” approaches could be focused on the tissues identified here as targets for remodeling and surely will allow the identification of differentially expressed genes involved in the process, which likely will serve to validate the present observations. These analyses will also serve to identify whether such differentially expressed genes are specific to drought, or common with other types of stresses, helping to find both already known or new players acting in this condition. Identification of factors that appear in both tissues under stress enables the further searching and testing of the substances that can modulate the activity of these compounds. Specific immunostainings here mentioned may be also used as assessing tools to assist in the selection of varieties and cultivars with enhanced tolerance to drought stress within breeding programs. As a consequence, it could improve the tolerance of crops to drought—the greatest problem for 21st-century plant breeding.

## 4. Materials and Methods

### 4.1. Plant Material and Growth Conditions

The experiments were performed on yellow lupine (*Lupinus luteus* L.). Seeds of taper cv. provided by Poznan Plant Breeding (Tulce, Wiatrowo, Poland) were sown and the plants were cultivated under the controlled conditions described by Frankowski et al. [3]. Lupines were watered for 5 weeks and during this time water holding capacity (70% WHC) was maintained [105]. After that, some of the plants were subjected to conditions of 25% WHC, while control plants were kept watered at 70% WHC. All analyses were made on root nodules and flower AZ fragments (Supplementary Figure S1) collected on the 48th day of development. We considered anatomical and morphological criteria described in our previous papers regarding the drought-dependent response of nodules and flower AZ [4,5]. For determination of proline, roots were divided into the upper, lower part, and nodules, which were excised gently with a razor blade under a binocular microscope. For the remaining analyses, only nodules were used. In turn, flower AZs were dissected precisely 1 mm above the flower bases (the distal AZ—a fragment of pedicel) and 1 mm below (the proximal part of the AZ—a fragment of stem). The experimental variants include nodules and AZ fragments from control (well-watered plants) and drought-stressed lupines (25% WHC). As we have recently shown, drought is a factor causing specific changes in nodules that affect their physiological activity–nitrogen fixation [4]. Furthermore, a water deficit in soil activates flower AZ, which leads to organ separation [5].

### 4.2. Determination of Proline and Pectin Content 

Proline was determined using the modified methodology of Ábrahám et al. [106]. All chemicals were provided by Sigma-Aldrich, St. Louis, MO, USA. Plant tissue (~0.5 g) was homogenized in 3% sulfuric acid (5 μL/mg fresh weight). The obtained extract was centrifuged at 18,000× *g* (5 min.). Subsequently, 100 μL of supernatant was mixed with 0.1 mL of 3% sulfosalicylic acid, 0.2 mL of glacial acetic acid, and 0.2 mL of acidic ninhydrin. The reaction mixture was further incubated at 96 °C for 30 min and then cooled on ice. After that, samples were mixed with 1 mL of toluene by vortexing. The absorbance of the organic phase was read at 520 nm. Proline concentration was calculated in reference to a calibration curve and expressed as μg proline g^−1^ fresh weight. Pectins were analyzed following the protocol of Liu et al. [107] which has been recently applied by us in lupine [8].

### 4.3. Protein Isolation, Dot Blot Assay, and Western-Blotting

Plant material (~0.4 g) was ground with liquid nitrogen and the obtained powder was mixed with 1 mL of extraction buffer (50 mM Tris-HCl buffer, pH 8.0, 300 mM NaCl, 10% *v*/*v* glycerol, and 1 mM EDTA). Subsequently, the samples were centrifuged at 4 °C for 10 min and the supernatant was frozen (−20 °C) for the next steps. The total pool of proteins was assessed by the Bradford method [108] and was adjusted to 0.5 µg/µL with extraction buffer. Crude protein extract was used for dot blot analysis. In brief, 5 µL of extract (2.5 µg proteins) were loaded in duplicate directly onto nitrocellulose membranes (Protran BA 83, Whatman GmBH), which were further blocked with 1.5 % bovine serum albumin (BSA) in TBS buffer pH 7.5 (150 mM NaCI, 20 mM Tris). After that, membranes were washed for 10 min with TBS (3 times). Then primary, monoclonal rat antibodies (JIM5, JIM7, LM5, LM6, LM8, LM24) provided by PlantProbes (Univeristy of Leeds, Leeds, UK) in 1:100 dilutions in TBS were served at room temperature for 24 h. The membranes were then washed 3 times (10 min. each) in TBS and subsequently incubated at room temperature in chicken anti-rat IgG, horseradish peroxidase (HRP) conjugated (AS101224, Agrisera, Vännäs, Sweden) in 1:10,000 dilutions in TBS. Epitopes were detected with Agrisera ECL SuperBright (AS16, Agrisera, Vännäs, Sweden) and scanned with a ChemiDoc™ Touch Imaging System.

For Western blot analysis, 30 µg of denatured proteins were separated on 4–12% (*w*/*v*) polyacrylamide gels (SDS-PAGE) in duplicate. After electrophoresis, one gel was stained with Coomassie Brilliant Blue while the second was subjected to electroblotting to a nitrocellulose membrane. To prevent the nonspecific binding of antibodies, the membrane was blocked with 1% BSA (Sigma-Aldrich, St. Louis, MO, USA) solution in TBS buffer pH 7.5 for 1 h at room temperature. Hemoglobin was detected using AHB2 antibody (AS132745, Agrisera, Vännäs, Sweden) diluted 1:1000 in 0.5% BSA in TBS buffer. The bound antibodies were detected with polyclonal secondary anti-rabbit antibody HRP diluted 1:10,000 in TBS buffer. The obtained protein complexes were detected using the Western Blot ECL kit (Agrisera, Vännäs, Sweden). Reactions were performed three times (*n* = 3) and each membrane was scanned for densitometry analysis. 

### 4.4. Immunohistochemistry

For immunocytochemical reactions, the plant tissue fragments were fixed using a multi-step procedure. All chemicals were provided by Sigma-Aldrich, St. Louis, MO, USA. Fresh material was placed immediately in a fixative solution composed of 4% paraformaldehyde, 0.25% glutaraldehyde in 1× PBS buffer (pH 7.2) with the addition of 1% 1-ethyl-3-(3ʹ-dimethylaminopropyl)carbodiimide and incubated for 12 h at 4 °C according to [109]. Next, tissues were rinsed, dehydrated, supersaturated with BMM resin, and polymerized as described in [109] and [8]. The material was then sealed in BEEM capsules and polymerized at low temperature (−20 °C) under a UV lamp for 4 days. The tissue was cut into semi-thin sections (approx. 1.5 μm) using an ultramicrotome (Leica UTC Ultracut Microtome, Leica, Wetzlar, Germany) and placed on glass slides. After that, the resin was removed with acetone twice for 10 min then in water and in PBS pH 7.2. For general structural observations, sections were stained with 0.05% toluidine blue (Sigma-Aldrich, St. Louis, MO, USA), while un-esterified pectin tissues were stained with 0.02% (*w*/*v*) ruthenium red (Sigma-Aldrich, St. Louis, MO, USA) solution, which we have applied previously in lupine [110].

### 4.5. Immunolocalization of Cell Wall Components

Selected plant cell components were visualized by immunocytochemical reactions on resin-free sections with specific primary antibodies provided by Agrisera (AHB2, Cat. No. AS132 745) or PlantProbes (Univeristy of Leeds, Leeds, UK), as follows: JIM5 (Cat. No. JIM5), JIM7 (Cat. No. JIM7), JIM11 (Cat. No. ELD030), JIM20 (Cat. No. ELD033), JIM8 (Cat. No. ELD024), JIM13 (Cat. No. JIM13-050), LM5 (Cat. No. LM5-050), LM6 (Cat. No. LM6-050), LM8 (Cat. No. LM8), LM24 (Cat. No. LM24). Antibodies were diluted (1:20) in 1% BSA in 1× PBS buffer (pH 7.2). The sections were incubated overnight at 4 °C. Next, sections were washed 3 times with 1× PBS buffer (pH 7.2) and incubated with secondary antibodies (goat anti-rat conjugated with FITC, Abcam, Cambridge, UK; goat anti-rabbit IgG DyLight 488 conjugated against AHB2, AS09 633, Agrisera, Vännäs, Sweden), 1:500 diluted in 1× PBS buffer. All reaction steps were conducted following our optimized protocol [109]. DNA was stained with 4,6-diamidino-2-phenylindole (DAPI) (Sigma-Aldrich, St. Louis, MO, USA) according to the producer′s recommendation. The results were documented using a fluorescent microscope DM6000B (Leica Microsystems GmbH, Wetzlar Germany). Control reactions were performed by omitting the incubation step with primary antibodies (Supplementary Figure S2). 

## Supplementary Materials

The following are available online at https://www.mdpi.com/article/10.3390/ijms23031680/s1
