# Supplementary material for: Remodeling of Cell Wall Components in Root Nodules and Flower Abscission Zone under Drought in Yellow Lupine
Source: Int J Mol Sci. 2022 Jan 31;23(3):1680. doi: 10.3390/ijms23031680 (PMC8836056; doi:10.3390/ijms23031680)
Supplement: Supplementary file 1 [file ijms-23-01680-s001.zip › ijms-1544567-supplementary.pdf]

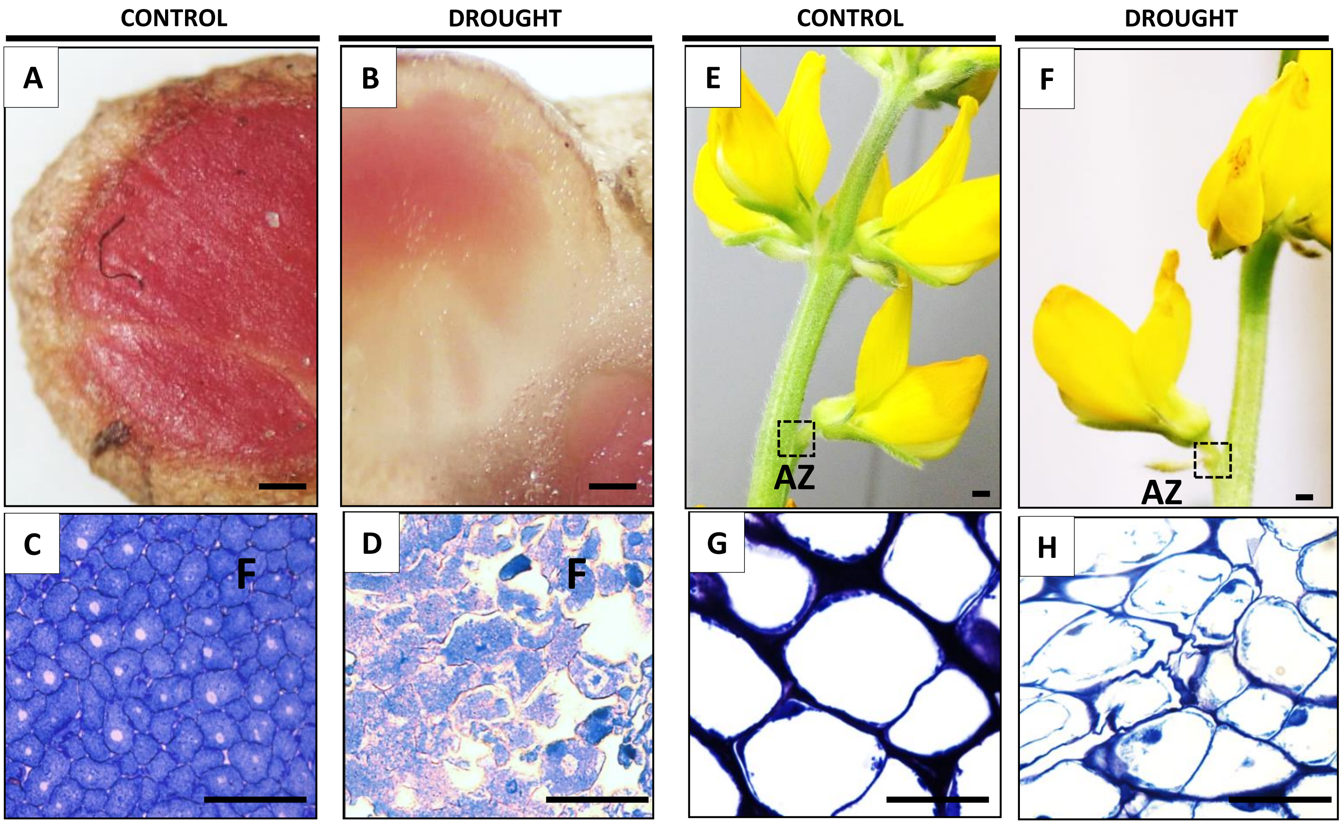

Figure S1. Histological analyses of nodules and flower abscission zone (AZ) of *Lupinus luteus.* Nodules were excised from plants cultivated for 2 weeks in soil drought (25% water holding capasity, WHC) (B) and under optimal conditions (70% WHC) (A). In paralel, flower AZ was harvested from the same, stressed (F) and control (E) lupines. Sections of control (C) and stressed (D) nodules stained with toluidine blue. Toluidine blue-stained sections of AZ from control (G) and stressed (H) plants. Abbreviations: AZ – abscission zone, F – fixation zone. Bar – 200 μm (A, B), 25 μm (C, D), 5 mm (E, F), 40 μm (G, H).

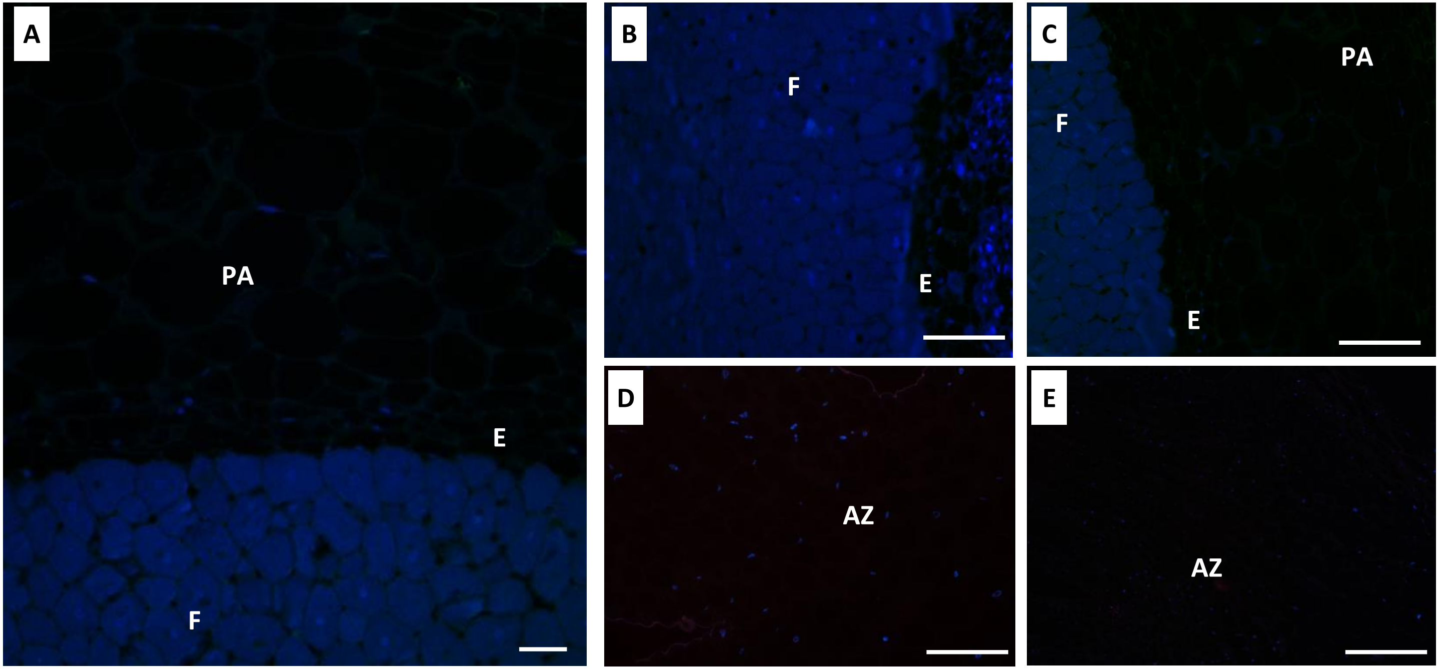

Figure S2. The results of control immunofluorescent reactions performed with omitting primary antibodies. Controls were performed using secondary antibodies only. Rabbit IgG DyLight 488 conjugated (Agrisera, Sweden) (A), Alexa Fluor 488 conjugated anti-rat IgG (Agrisera, Sweden) (B), goat anti-rat conjugated with FITC (Abcam, Cambridge) (C). Abbreviations: F – fixation zone; E – endodermis; PA – nodule parenchyma; AZ – abscission zone. Bar – 100 μm (A, B, C), 100 μm (D, E).
